# Effect of Excessive Body Weight and Psoriasis in Women Undergoing ICSI Procedure and State of Health of the Newborn

**DOI:** 10.3390/jcm9113628

**Published:** 2020-11-11

**Authors:** Anita Wdowiak-Filip, Artur Wdowiak, Dorota Raczkiewicz, Joanna Bartosińska, Iwona Bojar

**Affiliations:** 1 Department of Dermatology, Venerology and Pediatric Dermatology, Medical University of Lublin, ul. Radziwiłłowska 13, 20-080 Lublin, Poland; anita.wdowiak@gmail.com; 2 Diagnostic Techniques Unit, Medical University of Lublin, ul. Staszica 4/6, 20-081 Lublin, Poland; wdowiakartur@gmail.com; 3 Institute of Statistics and Demography, Collegium of Economic Analysis, SGH Warsaw School of Economics, ul. Madalińskiego 6/8, 02-513 Warszawa, Poland; 4 Department of Cosmetology and Aesthetic Medicine, Medical University, Chodzki 1, 20-093 Lublin, Poland; jbartosinski@gmail.com; 5 Institute of Rural Health in Lublin, ul. Jaczewskiego 2, 20-090 Lublin, Poland; iwonabojar75@gmail.com

**Keywords:** obesity, overweight, psoriasis, infertility, ICSI, pregnancy, APGAR, pregnancy complications

## Abstract

Excessive body weight and some concomitant diseases, such as psoriasis, accompany women treated due to infertility by intracytoplasmic sperm injection (ICSI). This study is aimed to assess effect of obesity and psoriasis on quality of egg cells, embryos, course of pregnancy, and state of a newborn after treatment with ICSI. A total of 140 women were included into the study (110 healthy women and 30 with psoriasis). Among healthy women, BMI negatively correlated with total recovery rate, total oocyte score, blastocyst formation rate (BFR) and amount and quality of blastocysts (r < 0, *p* < 0.001). The relationships were similar in psoriasis, however apart from average blastocyst quality (*p* = 0.17) and BFR (*p* = 0.352). In healthy patients, BMI negatively correlated with gestational age at delivery (r = −0.444, *p* = 0.010) and APGAR (r = −0.481, *p* = 0.005). An excess of adipose tissue exerts an unfavourable effect on female reproductive functions, especially with a simultaneous burden of psoriasis. Excessive body weight is conducive to development of gestational diabetes and shortens the duration of pregnancy. The burden of psoriasis in combination with excessive body weight has an impact on the risk of occurrence of intrauterine growth restriction of foetus. Overweight and obesity negatively affect the state of a newborn, measured using APGAR scale.

## 1. Introduction

An increasing number of people at reproductive age have difficulties achieving pregnancy. Therefore, infertility has been considered by the World Health Organization as a social disease [1]. For some couples, the only solution to this problem becomes the method of in vitro fertilization (IVF). However, the effectiveness of this method depends on various factors, such as overweight, or other concomitant diseases [1,2].

Over the past decades, an epidemic increase in the prevalence of overweight and obesity has become a worldwide problem [3], which results in a longer time to conceive and lower fertility. It is known that maternal obesity exerts a consequential unfavourable effect on the development of the preimplantation embryo. Moreover, early perturbations of the embryo may affect the health, not only of the offspring, but also of future generations [4]. Thus, obesity in pregnancy is related with negative clinical outcomes concerning the mother and foetus [5]. 

Apart from obesity, problems with conceiving and the course of pregnancy may also be complicated by concomitant diseases including, among others, psoriasis. Psoriasis, a chronic inflammatory skin disease with a complex pathogenesis consisting of a genetic component, immune dysfunction, and environmental factors, affects 2–4% of the general population.

According to various researchers, the prevalence of obesity in psoriasis is 15.8–20.7% [6]. Many epidemiologic studies confirm the relationship between obesity and psoriasis and, at present, it is considered that obesity is an independent factor of the development of this disease and is associated with worse prognosis.

Visceral adipose tissue is the largest endocrine organ producing pro-inflammatory cytokines and adipokines which participate in the development of dyslipidaemia, insulin resistance, diabetes, as far as the development of the metabolic syndrome and cardiovascular diseases. A special role is attributed to the visceral adipose tissue macrophages, the number of which considerably increases in obesity. They are responsible for the development of inflammatory condition in the adipose tissue and production of pro-inflammatory cytokines (tumour necrosis factor α (TNFα), IL-6, -8, -17, -18, monocyte chemoattractant protein-1 (MCP-1)), as well as the remaining adipokines: resistin, visfatin, retinol-binding protein 4. This provides an explanation to the concept of the “psoriatic march” and frequently observed co-occurrence of psoriasis and obesity. In psoriasis, systemic inflammation, enhanced by pro-inflammatory cytokines and adipokines, induces insulin resistance and damages the endothelial cells. Endothelial dysfunction predisposes to the development of atherosclerotic plaques and quicker development of cardiovascular events [6,7].

Recent studies revealed that some immune-mediated inflammatory conditions, i.e., rheumatoid arthritis and inflammatory bowel diseases, especially in their active stages, can impact female fertility and pregnancy outcomes [8,9]. Thus, considering similar pathogenesis in autoimmune diseases, autoimmunity in psoriasis may be one of the causes of diminished ovarian reserve [10].

In psoriasis, hormonal changes, metabolic abnormalities and systemic inflammation could influence the ability to conceive, pregnancy course and birth outcomes [10].

It may be presumed that a generalized inflammatory condition related with psoriasis and obesity may exert an effect on the reproductive functions of a woman through its impact on the environment of maturation of the egg cell. Due to reproductive problems, some women qualify for an in vitro fertilization procedure. During the procedure, it is possible to evaluate the oocytes and embryos; however, this problem has not yet been investigated in women with psoriasis.

The aim of the present study was assessment of the effect of obesity and psoriasis on the quality of egg cells, embryos, course of pregnancy, and the state of a newborn after treatment with an in vitro fertilization method.

## 2. Material and Methods

### 2.1. The Study Patients

The presented retrospective single centre case-control study cohort study was conducted in 2018–2020 in the “Ovum Reproduction and Andrology” Non-Public Health Care Unit in Lublin, Poland, among women treated for intracytoplasmic sperm injection (ICSI). Before in vitro fertilization, patients were informed about the effectiveness of ICSI and the classic IVF, and could choose one of the methods. Considering the higher effectiveness of ICSI, the patients decided to choose this method.

The study was comprised of 30 female patients with mild plaque psoriasis and 110 age-matched female patients without psoriasis who underwent ovarian stimulation. The psoriasis women and women without psoriasis were interviewed and subjected to a physical examination, including the measurement of body weight and height. Body mass index (BMI) was calculated in all the women in the study.

Informed consent was obtained from each participant. The study protocol complied with the ethical guidelines of the 1975 Declaration of Helsinki, and was approved by the local Ethics Committee.

The study was approved by the Medical Ethics Committee in Medical University of Lublin, number KE-0254/48/2019.

### 2.2. Characteristics of Psoriasis in the Study Patients

Psoriasis was diagnosed by a dermatologist on the basis of patient’s history and the clinical signs. The severity and extent of psoriasis were assessed using Psoriasis Area and Severity Index (PASI), Body Surface Area (BSA) and Dermatology Life Quality Index (DLQI), a 10-item questionnaire with scores ranging from 0–30. In order to calculate PASI (range 0–72), the severity of three clinical signs: erythema (redness), induration (thickness) and desquamation (scaling) was estimated within 4 areas of the skin (head, arms, trunk, and legs).

Psoriasis was defined as mild when psoriatic skin lesions covered less than 10% of the total body surface (BSA < 10%), the severity of skin lesions expressed by PASI was <10 and the negative impact of the disease on the patient’s quality of life assessed by DLQI was <10.

The inclusion criteria for the psoriasis women were mild psoriasis, plaque type, type 1 psoriasis (onset before 40 years old). The exclusion criteria for the psoriasis women: moderate-to-severe psoriasis, other types of psoriasis, i.e., erythrodermic, pustular, psoriatic arthritis, type 2 psoriasis (onset after 40 years old), systemic treatment three months prior to the study, topical anti-psoriatic drugs two weeks prior to the study.

### 2.3. Characteristics of the Study Patients

Women aged 30–35 who had undergone intracytoplasmic sperm injection (ICSI) procedure for the first time, considering only autologous cycles were enrolled into the study. All women participating in the study had ovulatory cycles, and the patency of at least one fallopian tube was confirmed. The parameters of the semen of their partners were normal according to WHO 2010. In the past, the women in the study had problems with becoming pregnant, which were associated especially with multifollicular growth after monofollicular stimulation in three subsequent cycles, follicles rupture, or three insemination procedures failed. 

The criteria for exclusion of all women participating in the study were as follows: diagnosis of insulin resistance (IR) or hyperglycaemia, ovulatory dysfunction due to pathologic conditions (endometriosis, hyperprolactinaemia, hypothyroidism), excess amounts of androgens (adrenal hyperplasia, Cushing’s syndrome), Polycystic Ovary Syndrome (PCOS), diagnosed according to the Rotterdam ESHRE–ASRM Sponsored PCOS consensus workshop group. 

Women were divided into 4 groups according to the World Health Organization (WHO) classification cut-off points:

1. Healthy women with normal-weight (18.5–24.9 kg/m^2^) (N = 55);
2. Healthy women with overweight (25–29.9 kg/m^2^) or Class I obesity (30–34.9 kg/m^2^) (N = 55);
3. Normal weight with psoriasis (N = 15);
4. Overweight or Class I obesity with psoriasis (N = 15).

Overweight and Class I obesity were grouped together due to the small number of women. In the infertility treatment centre, women with Class II obesity (BMI 35–39.9 kg/m²) and Class III obesity (BMI ≥ 40 kg/m^2^) were not qualified for ICSI until the reduction of weight down to Class I obesity.

### 2.4. The Study Protocol

Luteal gonadotropin-releasing hormone analogue (GnRHa) (Gonapeptyl Daily^®^: Ferring GmbH, Kiel, Germany) and, subsequently, recombinant FSH (Bemfola^®^: Gedeon Richter Plc, Budapest, Hungary) were applied for ovarian stimulation from the third day of the cycle in a short protocol. While performing ovarian stimulation, the number of follicles (17 mm) and endometrial thickness (mm) were assessed by means of the Voluson E8 Expert. On the day of rhCG administration, a morning blood sample (5 mL) was taken and serum levels of estradiol (E2) (pg/mL) were evaluated. On day 3 of the cycle preceding ovulation, the level of AMH (ng/mL) was analyzed in an authorized laboratory. 

Thirty-six hours after application of recombinant human chorionic gonadotropin administration (rhCG) (Ovitrelle^®^: Merck Serono, Feltham, UK), vaginal ultrasound-guided aspiration of oocyte-cumulus complexes was carried out. A standard procedure was adopted in the study; in cases where, on the day of application of rhCG administration, the level of progesterone was higher than 1.2 ng/mL, the transfer would not be performed, and all the embryos would be frozen. In all patients, on the day of application of rhCG administration, the level of progesterone was lower than 1.2 ng/mL. 

Three hours after retrieval oocyte denudation and ICSI were carried out, and subsequently in vitro culture was performed in 25 μL of Cleavage medium (COOK, Sydney IVF, Australia) under mineral oil until day 2 (2–5 cells stage) in automated incubators with 5% CO_2_ at 37 °C. Fifty hours following ICSI the in vitro culture media was changed to blastocyst medium (COOK, Sydney IVF, Australia).

### 2.5. Oocyte Quality

Six characteristics of egg quality were determined: morphology, cytoplasm [11,12,13,14,15,16,17,18,19], perivitelline space (PVS) [20,21], zona pellucida (ZP) [22,23], polar body (PB) [24,25], and oocyte size [26], each assigned a value of −1 (worst), 0 (average) or +1 (best), thus determining an average total oocyte score (TOS).

Only intracytoplasmic sperm injection cycles and patients undergoing embryo transfer on day 5 (blastocyst) took part in the study. 

### 2.6. Blastocyst Quality

In order to differentiate between embryos with “excellent”, “good”, “average”, and “poor” quality, based on the parameters of the blastocysts, the expansion, trophectoderm (TE), and inner cell mass (ICM) were evaluated.

First, a numeric score (1–6) was assigned based on their degree of expansion and hatching status: (1) early blastocyst (i.e., the blastocoel that is less than half of the volume of the embryo); (2) blastocyst, (the blastocoel occupying more than half of the volume of the embryo); (3) full blastocyst, (the blastocoel completely filling the embryo); (4) expanded blastocyst, (the blastocoel volume being larger than that of the early embryo, and the zona starting to thin); (5) hatching blastocyst, (trophectoderm stars herniating though the zona pellucida); and (6) hatched blastocyst (the blastocyst has escaped completely from the zona pellucida). For blastocysts graded 3–6 (i.e., full blastocysts onward), the development of the ICM and TE were subsequently evaluated. The ICM was assigned one of the 3 grades: A, numerous tightly packed cells; B, loosely grouped, with several cells; and C, very few cells. The TE grading was as follows: A, many cells forming a cohesive epithelium; B, few cells, forming a loose epithelium; and C, very few large cells [27].

For the purpose of comparison of the results of the present and previous studies by Capalbo et al. [28] and Irani et al. [29], immediately before performing biopsy, the blastocysts were assigned a three-character score according to their degree of expansion, and ICM and TE grades. These scores ranged as follows:

1. Excellent (≥3AA);
2. Good (3–6AB, 3–6BA, 1–2AA);
3. Average (3–6BB, 3–6AC, 3–6CA, 1–2AB, 1–2BA);
4. Poor (1–6BC, 1–6CB, 1–6CC, 1–2BB) grade blastocysts.

Two weeks after the punction, the hCG level was evaluated to discover if the woman was pregnant and, after week 6 of pregnancy, ultrasound examination was performed to assess the presence of an embryo and cardiac activity. Information was collected pertaining to complications during pregnancy, such as 1-gestational diabetes (GDM), 2-gestational hypertension (GHT), 3-gestational proteinuric hypertension (PET), 4-intrauterine growth restriction (IUGR), week of pregnancy when birth occurred, and the state of the newborn after birth measured using the APGAR scale after 5 min [30]. As the main aim of the study was to assess the state of the newborn, women who had a miscarriage were excluded from the study and only live births were analysed. There was no intrauterine foetal death among the examined women.

### 2.7. Statistical Methods

The data were statistically analysed using the STATISTICA 13 software. The mean and standard deviation were estimated for continuous variables, as well as absolute numbers (n) and percentages (%) of the occurrence of items for categorical variables.

The following statistical tests were used:

- Mann–Whitney U test to compare numerical variables: in healthy women between normal weight and overweight or Class I obesity, in psoriatic women between normal weight and overweight or Class I obesity, in healthy women with overweight or Class I obesity versus psoriatic women with overweight or Class I obesity, in healthy women with normal weight versus psoriatic women with normal weight;
- Pearson’s chi-square test to compare the categorical variables in healthy women between normal weight and overweight or Class I obesity, in psoriatic women between normal weight and overweight or Class I obesity;
- Pearson’s correlation coefficient to correlate BMI with numerical variables.

The significance level was assumed to be 0.05 in all statistical tests.

## 3. Results

Table 1 presents results concerning the course of ICSI procedure in individual groups. Age (approximately 32 years on average) and serum AMH concentration (approximately 3 pmol/L on average) did not significantly differ between healthy women with normal weight, healthy women with overweight or Class I obese, psoriatic women with normal weight and psoriatic women with overweight or Class I obesity (*p* > 0.05).

In women with overweight or Class I obesity, higher doses of gonadotropins were needed for stimulation (*p* < 0.001), and a lower level of estradiol was obtained, compared to women with normal weight (*p* < 0.001). After stimulation, a similar amount of follicles was obtained in all groups (approximately 8 in one patient); however, the total recovery rate was significantly decreased in women with overweight or Class I obesity (*p* < 0.001). Additionally, TOS, the amount and quality of blastocyst, as well as BFR, were significantly decreased in women with overweight or Class I obesity (*p* < 0.001). The mentioned above differences concerned both the healthy women and the psoriatic women.

In Table 1 we also compared the course of ICSI procedure between healthy women with overweight or class I obesity and psoriatic women with overweight or class I obesity. In the psoriatic women with excessive body weight we observed significantly lower: number of oocytes retrieved (*p* = 0.006), total recovery rate (*p* = 0.001), average TOS of oocytes developed to transferred blastocysts (*p* = 0.003) and BFR (*p* = 0.016) in comparison to healthy women with excessive body weight.

Then in Table 1 we compared the course of ICSI procedure between healthy women with normal weight and psoriatic women with normal weight. The psoriatic women with normal weight had obtained significantly higher dose of total amount of rFSH than the healthy women with normal weight. We did not find any statistical differences in the course of ICSI procedure between the healthy women with normal weight and the psoriatic women with normal weight.

Table 2 presents the percentage of live births, occurrence of pregnancy complications and gestational age in individual groups. Among healthy women with normal weight, 36.36% of live births were achieved, whereas 23.64% among women overweight or Class I obese (*p* = 0.14). Similarly, 40% of live births were achieved in psoriatic women with normal weight and 13.33% among those overweight or Class I obese (*p* = 0.09). Overweight women with psoriasis might have poorer response to ICSI; nevertheless, the pregnancy and live birth rates were no different from the control group. Among healthy women, gestational diabetes occurred significantly more often in overweight or Class I obese patients, compared to those with normal weight (*p* = 0.039), and a similar relationship concerned women with psoriasis (*p* = 0.005). In the group with psoriasis, IUGR was more rarely observed in women with normal weight (*p* = 0.034). The time of delivery of pregnancy was significantly higher in healthy women with normal weight than in those overweight or Class I obese (*p* = 0.006), while in the group with psoriasis no differences were observed in this term (*p* = 0.24).

In Table 2, we also compared the percentage of live births, occurrence of pregnancy complications and gestational age between healthy women with overweight or class I obesity and psoriatic women with overweight or class I obesity. In psoriatic women with excessive body weight, we observed significantly higher prevalence of gestational diabetes (*p* = 0.008) and intrauterine growth (*p* = 0.021) in comparison to healthy women with excessive body weight.

Then, in Table 2, we compared the percentage of live births, occurrence of pregnancy complications and gestational age between healthy women with normal weight and psoriatic women with normal weight. In psoriatic women with normal weight, we observed significantly higher prevalence of intrauterine growth in comparison to healthy women with normal weight (*p* < 0.001).

APGAR < 7 was observed in newborns of 50% of the psoriatic women with overweight or Class I obesity and 16.7% of the psoriatic women with normal weight, but was not observed in newborns of healthy women with normal weight or healthy women with overweight or Class I obesity.

Table 3 presents correlations between BMI and parameters associated with the course of ICSI procedure and pregnancy termination. Among healthy women, BMI negatively correlated with total recovery rate, TOS, BFR and the amount and quality of blastocysts (r < 0, *p* < 0.001). In the group with psoriasis the relationships were similar; however, they did not concern the average blastocyst quality (*p* = 0.176) and BFR (*p* = 0.352).

In healthy patients, BMI negatively correlated with gestational age at delivery (r = −0.444, *p* = 0.010) and APGAR (r = −0.481, *p* = 0.005). 

## 4. Discussion

Although pregnancy and live birth rates are no different, the current study shows that an excessive body weight exerts an unfavourable effect on the course of treatment using the ICSI procedure, through quantitative and qualitative disorders in the production of gametes, which negatively affects the quality of the embryos obtained. These observations are in accordance with reports by many researchers published in recent years [31,32]. Nevertheless, there are studies which postulate the lack of relationship between BMI and the course of the ICSI procedure [33]. The study participants with normal body weight needed lower doses of gonadotropines to stimulate ovulation, compared to women with excessive body weight, which has been confirmed in reports by other researchers [34,35].

The percentage of achieved pregnancies in the groups did not significantly differ statistically, which may result from the small number of examined women with psoriasis, and consideration in the criteria of inclusion into the study the patients with exclusively mild form of the disease. A higher percentage of pregnancies achieved in the group of women with normal body weight, both with and without psoriasis, may be associated with a better quality of oocytes and blastocysts. The lowest percentage of pregnancies was achieved in the group of obese women with psoriasis. This may be related to decreased fertility in women with psoriasis described by several researchers [10,36]. This undoubtedly requires analysis in a larger study group. However, psoriasis affects 2–4% of the population, which hindered the collection of a group of comparable size [37].

Women with moderate-to-severe psoriasis in Spain showed a more than 50% reduction in age-adjusted fertility rate when compared with the general population, which is comparable with the effectiveness of ICSI procedure performed in the presented study in a group of 30 women with a mild form of plaque psoriasis. This fact may be explained in a study by Tuğrul Ayanoğlu B. et al. [38], who observed a lower ovarian reserve in patients with psoriasis, compared to their healthy contemporaries. Similar observations were described by Aydogan Mathyk B. et al., who additionally found that the duration of the disease negatively correlated with AMH, total AFC and ovarian volume [39].

Moderate-to-severe psoriasis patients are commonly treated with conventional systemic anti-psoriatic drugs (i.e., methotrexate, cyclosporine, retinoids) which may influence conception and pregnancy outcome. A faster diminishing of the ovarian reserve may also be related with the fact of using methotrexate during treatment of psoriasis. In animal studies, methotrexate showed teratogenicity, embryo death, low birth weight and congenital malformations. Due to the lack of mutagenicity and no increased risk of congenital malformations, cyclosporine is used in severe psoriasis in pregnancy. However, some studies on cyclosporine post-organ transplantation confirmed an increased risk of premature birth and low birth weight in the infants [40,41]. In animal studies, cyclosporine doses negatively correlated with the implantation rate, suggesting its detrimental effect on maternal fertility [42]. The well-known high risk of teratogenic effects associated with retinoids requires the use of contraception for 3 years after cessation of the treatment. 

In the treatment of psoriasis, other drugs (i.e., biologics and a new class of small-molecule drugs) are also applied which may not be neutral for reproductive capabilities. Although the potential impact of biologics and emerging therapies for psoriasis on future fertility is reassuring for patients, more data are needed [43]. 

Kong et al. analyzed the influence of biologics and emerging anti-psoriatic therapies on fertility [43]. The limited available human data on adalimumab and etanercept did not reveal gonadal toxicity. Animal studies in infliximab, ixekizumab, secukinumab, ustekinumab and brodalumab did not show ovarian toxicity with testing performed 8–50 times beyond the maximum recommended human dose, and were classified in category B. [44]. Interestingly, biologic agents appeared to improve fertility, especially when gonadal impairment was related to inflammatory phenomena [45].

In animal studies, apremilast—at 1.8 times the maximum recommended human dose—caused prolonged estrous cycles and increased early postimplantation losses. Since phosphodiesterase 4 degrades, vital for egg maturation, cyclic adenosine monophosphate, apremilast could affect fertility. The janus kinase inhibitors (tofacitinib, ruxolitinib) resulted in increased post-implantation losses in mice. Higher doses of tofacitinib resulted in decreased pregnancy rate, corpora lutea, implantation sites, and viable foetuses. Moreover, janus kinase signalling plays a critical role in the neuroendocrine control of female reproduction in mice [43,46,47]. 

In our study, we excluded patients with moderate-to-severe psoriasis, and patients treated with systemic anti-psoriatic drugs, in order to eliminate their potential impact on the effectiveness of the ICSI procedure [48,49].

In the conducted study, the greatest decrease in the reproductive capabilities was observed in the group of obese women with psoriasis. Many epidemiological studies confirm the relationship between obesity and psoriasis, and at present obesity is considered an independent factor of its development and is associated with worse diagnosis. Bandoli et al., in a group of pregnant women, observed that those with psoriasis were 2.37 (95% confidence interval 1.45–3.87) times more likely to be overweight/obese than women without psoriasis [50].

Psoriasis and obesity share common pathogenic mechanisms, i.e., increased production of pro-inflammatory cytokines (IL-1, IL-6, TNF-α, and adiponectin) which are responsible for a chronic low-grade inflammation which are observed in both conditions [51]. In the case of this group of women, the most intensified disorders of the oxidation reduction system may be expected. The environment in which the egg cell maturates has an impact on its quality, as well as the quality of the embryo. Adipose tissue accumulated in a woman’s body contributes to an increase in the phenomenon of oxidative stress by the production of an excess of adipocytokines and reactive oxygen species. At present, the white adipose tissue is defined as the largest, metabolically active endocrine organ of the body. Cells of the white adipose tissue (adipocytes) have the capability to produce and secrete many biologically active substances called adipocytokines. Adipocytokines formed and released by the cells of adipose tissue may be responsible for a chronic, sub-clinical inflammatory state leading to damage to the reproductive cells. 

Additionally, in psoriasis, regardless of the disease severity, increased oxidative stress, adipokine imbalance and impaired endothelial function are observed. In patients with psoriasis, elevated levels of leptin, chemerin, visfatin and resistin have been found, with decreased levels of protective adipokines: adiponectin and omentin [52]. 

The presented study demonstrated that gestational diabetes more frequently occurred among obese pregnant women. This observation has been confirmed by other researchers [53,54]. An excessive body weight is a risk factor of the development of gestational diabetes.

In obese patients with psoriasis in our study, intrauterine growth restriction (IUGR) occurred more often than in those with a normal body weight. There are several studies concerning the relationship between psoriasis and low birth weight of infants. The researchers emphasize the relationship between this complication and the activity of the disease [55]. The results of a Taiwanese study that included 1463 mothers with psoriasis and 11,704 controls revealed that women with severe psoriasis were at an increased risk of giving birth to low-birth-weight infants. Contrary to our results, the authors did not observe an excess risk in women with mild psoriasis. Nevertheless, obesity itself is the factor conducive to IUGR. The small number of patients with psoriasis participating in the study hindered a reliable analysis of this problem. 

The conducted study demonstrated an unfavourable effect of excessive body weight on APGAR. The results obtained are in accordance with meta-analysis by Zhu et al. which showed similar relationships after analysis of 11 cohort studies with a total of 2,586,265 participants [56]. APGAR is associated with delivery date, and in our study women with excessive body weight delivered earlier, compared to those with normal weight, which may explain the relationships observed.

A limitation of the study is a small sample size of 30 women with mild psoriasis who underwent ICSI procedure. However, such a sample size was satisfied for us since psoriasis is a rare disease which is diagnosed in 2–3% of population. Additionally, in the study, we included only women with mild psoriasis since most of the patients with moderate-to-severe stage of the disease is treated or have a history of systemic anti-psoriatic treatment which could affect implantation rate. Thus, achieving larger sample size in a single medical centre is limited. 

The presented study suggests that an excessive body weight, as well as the burden of psoriasis, exert an unfavourable effect on human reproductive functions as early as from the stage of gametogenesis, through conception, early embryonic stage and course of pregnancy, as well as the state of the newborn. However, the observations made require confirmation on a larger number of cases.

## 5. Conclusions

An excess of adipose tissue exerts an unfavourable effect on female reproductive functions, especially with a simultaneous burden of psoriasis. Excessive body weight is conducive to the development of gestational diabetes and shortens the duration of pregnancy. The burden of psoriasis in combination with excessive body weight has an impact on the risk of occurrence of intrauterine growth restriction of the foetus. Overweight and obesity negatively affect the state of health of a newborn, measured using the APGAR scale.

## Figures and Tables

**Table 1:** The course of intracytoplasmic sperm injection (ICSI) procedure in both healthy and psoriatic women with normal weight and overweight or Class I obesity. Data are presented as M ± SD.

Parameter	Healthy Women			Psoriatic Women			*p* ^3^	*p* ^4^
	Normal Weight (N = 55)	Overweight or Class I Obesity (N = 55)	*p* ^1^	Normal Weight (N = 15)	Overweight or Class I Obesity (N = 15)	*p* ^2^		
Age (years)	31.80 ± 1.89	31.87 ± 2.12	0.92	31.67 ± 1.35	31.67 ± 1.50	0.93	0.63	0.458
AMH (pmol/L)	3.03 ± 0.95	2.95 ± 0.83	0.58	3.27 ± 0.76	2.93 ± 0.82	0.19	0.98	0.44
Total amount of rFSH (IU)	1301 ± 187	1640 ± 343	**<0.001**	1370 ± 169	1790 ± 378	**0.001**	0.13	**0.047**
Endometrial thickness (mm)	11.71 ± 1.48	12.08 ± 2.05	0.24	12.47 ± 1.08	12.16 ± 2.20	0.98	0.84	0.06
E2 (pg/mL)	2379 ± 1461	1478 ± 1013	**<0.001**	1989 ± 483	1216 ± 382	**<0.001**	0.65	0.98
Number of follicles aspirated	8.27 ± 2.66	7.62 ± 2.97	0.06	7.60 ± 1.30	7.27 ± 1.53	0.47	0.94	0.50
Number of oocytes retrieved	7.73 ± 2.58	6.02 ± 2.31	**<0.001**	7.20 ± 1.37	4.60 ± 0.91	**<0.001**	**0.006**	0.68
Total recovery rate	0.94 ± 0.09	0.80 ± 0.15	**<0.001**	0.95 ± 0.07	0.65 ± 0.13	**<0.001**	**0.001**	0.88
Average TOS	2.45 ± 0.87	0.91 ± 0.96	**<0.001**	2.46 ± 0.82	1.33 ± 1.12	**0.007**	0.74	0.90
Average TOS of oocytes developed to transferred blastocysts	5.55 ± 0.57	3.89 ± 1.10	**<0.001**	5.57 ± 0.37	2.97 ± 0.83	**<0.001**	**0.003**	0.60
Number of frozen blastocysts	3.07 ± 2.16	0.64 ± 0.95	**<0.001**	2.47 ± 1.30	0.60 ± 0.74	**<0.001**	0.81	0.93
Number of all blastocysts (transferred + frozen)	4.93 ± 1.95	2.38 ± 1.18	**<0.001**	4.47 ± 1.30	2.47 ± 0.92	**<0.001**	0.56	0.56
BFR	0.64 ± 0.13	0.55 ± 0.23	**<0.001**	0.62 ± 0.12	0.40 ± 0.14	0.12	**0.016**	0.64
Average blastocyst quality	2.79 ± 0.79	2.00 ± 0.38	**<0.001**	2.37 ± 0.67	1.82 ± 0.21	**0.002**	0.11	0.07
Average quality of transferred blastocysts	2.61 ± 0.91	1.42 ± 0.53	**<0.001**	2.20 ± 0.68	1.23 ± 0.37	**<0.001**	0.15	0.27

*p*^1^—normal weight versus overweight or Class I obesity in healthy women. *p*^2^—normal weight versus overweight or Class I obesity in psoriatic women. *p*^3^—healthy women with overweight or Class I obesity versus psoriatic women with overweight or Class I obesity. *p*^4^—healthy women with normal weight versus psoriatic women with normal weight. *p* for Mann–Whitney’s U test. Significant differences are in bold. M—mean, SD—standard deviation, AMH—Anti-Müllerian hormone, rFSH—Human recombinant follicle stimulating hormone, E2—Oestradiol, Total recovery rate = oocytes retrieved/follicles aspirated, TOS—total oocyte score, BFR—blastocyst formation rate.

**Table 2:** Live births, complications during pregnancy and gestational age in both healthy and psoriatic women with normal weight and overweight or Class I obesity.

Parameter	Healthy Women			Psoriatic Women			*p* ^3^	*p* ^4^
	Normal Weight (N = 55)	Overweight or Class I Obesity (N = 55)	*p* ^1^	Normal Weight (N = 15)	Overweight or Class I Obesity (N = 15)	*p* ^2^		
Live births, n (%)	20 (36.36)	13 (23.64)	0.14	6 (40.00)	2 (13.33)	0.09	0.39	0.80
Complications during pregnancy, n (%)								
gestational diabetes	2 (3.64)	4 (7.27)	0.40	0 (0.00)	5 (33.33)	**0.005**	**0.008**	0.44
gestational hypertension	0 (0.00)	3 (5.45)	**0.039**	0 (0.00)	1 (6.67)	0.23	0.86	0.99
gestational protein uric hypertension	1 (1.82)	3 (5.45)	0.30	0 (0.00)	3 (20.00)	**0.034**	0.74	0.60
intrauterine growth	1 (1.82)	5 (9.09)	0.08	4 (26.67)	5 (33.33)	0.69	**0.021**	**<0.001**
Gestational age (weeks) at delivery, M±SD	38.85 ± 1.42	37.38 ± 1.71	**0.006**	37.33 ± 2.34	34.50 ± 0.71	0.24	0.07	0.11

*p*^1^—normal weight versus overweight or Class I obesity in healthy women. *p*^2^—normal weight versus overweight or Class I obesity in psoriatic women. *p*^3^—healthy women with overweight or Class I obesity versus psoriatic women with overweight or Class I obesity. *p*^4^—healthy women with normal weight versus psoriatic women with normal weight. *p* for Mann-Whitney’s U test or for chi-square test. Significant differences are in bold. M—mean, SD—standard deviation.

**Table 3:** Correlations of BMI (kg/m^2^) with age, AMH, the course of ICSI procedure, pregnancy termination and APGAR in healthy women, and in psoriatic women.

Parameter	Healthy Women (N = 110)		Psoriatic Women (N = 30)	
	r	*p*	r	*p*
Age (years)	0.045	0.64	0.028	0.88
AMH (pmol/L)	0.029	0.77	−0.250	0.18
Total amount of rFSH (IU)	0.437	**<0.001**	0.333	0.07
Endometrial thickness (mm)	0.060	0.53	−0.005	0.98
E2 (pg/mL)	−0.222	**0.020**	−0.596	**0.001**
Number of follicles aspirated	0.022	0.82	−0.043	0.82
Number of oocytes retrieved	−0.198	**0.038**	−0.638	**<0.001**
Total recovery rate	−0.471	**<0.001**	−0.703	**<0.001**
Average TOS	−0.581	**<0.001**	−0.422	**0.020**
Average TOS of oocytes developed to transferred blastocysts	−0.596	**<0.001**	−0.739	**<0.001**
Number of frozen blastocysts	−0.512	**<0.001**	−0.541	**0.002**
Number of all blastocysts (transferred + frozen)	−0.540	**<0.001**	−0.587	**0.001**
BFR	−0.647	**<0.001**	−0.176	0.35
Average blastocyst quality	−0.493	**<0.001**	−0.254	0.18
Average quality of transferred blastocysts	−0.556	**<0.001**	−0.414	**0.023**
Gestational age (weeks) at delivery	−0.444	**0.010**	−0.303	0.47
APGAR	−0.481	**0.005**	−0.356	0.39

r—Pearson’s correlation coefficient. Significant correlations are in bold. BMI—body mass index, AMH—Anti-Müllerian hormoner, rFSH—Human recombinant follicle stimulating hormone, E2—Oestradiol, Total recovery rate = oocytes retrieved/follicles aspirated, TOS—total oocyte score, BFR—blastocyst formation rate.
